# A comparison study to assess U-Net driven volumetric versus single-slice analysis and MRI sequences with different volume coverage to detect renal sinus fat in people with and without diabetes

**DOI:** 10.1038/s41598-025-33098-0

**Published:** 2026-02-03

**Authors:** Filippo C. Michelotti, Rio Koshiba, Clara Möser, Katharina S. Massold, Tim Mori, Yuliya Kupriyanova, Michael Roden, Robert Wagner, Vera B. Schrauwen-Hinderling

**Affiliations:** 1 Institute for Clinical Diabetology, German Diabetes Center, Leibniz Center for Diabetes Research at Heinrich Heine University Düsseldorf, Auf′m Hennekamp 65, 40225 Düsseldorf, Germany; 2 German Center for Diabetes Research (DZD), Partner Düsseldorf, München-Neuherberg, Germany; 3 Department of Endocrinology and Diabetology, Medical Faculty and University Hospital Düsseldorf, Heinrich Heine University, Düsseldorf, Germany; 4 Institute for Biometrics and Epidemiology, German Diabetes Center, Leibniz Center for Diabetes Research at Heinrich Heine University Düsseldorf, Düsseldorf, Germany

**Keywords:** MRI, Renal sinus fat, Renal parenchyma, Image segmentation, Diabetes, Diseases, Health care, Medical research, Nephrology

## Abstract

Monitoring the accumulation of renal sinus fat (RSF) by non-invasive magnetic resonance imaging (MRI) holds promise for assessing the risk of nephropathy in individuals with diabetes. Automatic image segmentation using dedicated U-Net models was deployed for accurate quantification of RSF content and renal parenchyma (RP) from different MRI protocols. Therefore, the accuracy of volumetric vs single-slice analysis for quantifying RP and RSF was assessed. Further, the resulting kidney structures obtained from a whole-body MR images acquired with partial kidney coverage were compared to high-resolution MRI protocol with full-kidney coverage, in people with and without diabetes. Quantification of kidney structures showed accurate estimates of both RP and RSF volume across people with different glycaemic status and imaging protocols. A systematic overestimation of the RSF-to-RP ratio was observed when using the conventional single-slice assessment, supporting the need for volumetric kidney analysis, particularly for small structures such as the RSF. Moreover, MR images with interslice-gaps were found to substantially underestimate RSF content, highlighting the need for careful evaluation and correction of estimates from small kidney structures when data are pooled from different MR imaging protocols. In summary, automatic image segmentation enabled us to determine differences in the precision of RSF content obtained using different methodological approaches and MRI sequences with different kidney coverage.

**Supplementary Information:**

The online version contains supplementary material available at 10.1038/s41598-025-33098-0.

## Introduction

Based on The Global Burden of Disease 2015 Study, the mortality of people with chronic kidney disease (CKD) due to diabetes has increased by 39.5% over the decade 2005–2015, reaching 418 thousand deaths, with a prevalence of CKD in type 2 diabetes (T2D) estimated to be 27% nowadays^1,2^.

Recent studies indicate that people with T2D have an accumulation of adipose tissue in the renal sinus, which was previously linked to hypertension and CKD onset^3,4^. The renal sinus contains various tissue types, including nerve fibers, adipose tissue, lymphatic veins and fibrous tissue, and can be indirectly affected by different diseases of the renal parenchyma^5^. Vice-versa, the accumulation of renal sinus fat could affect the renal function. In this context, assessing renal sinus fat (RSF) based on magnetic resonance imaging (MRI) is a promising marker for the non-invasive follow-up of metabolic disease progression and for the identification of people at risk for nephropathy^6,7^.

In common practice, single-slice image analysis was typically used to assess RSF content at the height of the renal pelvis^3,8,9^, which however can be affected by the heterogeneous distribution of this fat compartment across the kidney volume. Further, the quantification from whole-body MR images acquired with partial-kidney coverage and large slice thickness can be biased, especially for small and ill-defined regions such as the renal sinus fat. For this purpose, more recent advanced high-resolution protocols with full-kidney coverage and water-fat separation modules will be necessary.

For large volumes the lack of information is compensated by interpolating the areas throughout the acquired images. However, it remains unclear whether estimates of RSF obtained from such protocols with interslice gaps are comparable to high-resolution MR images with full kidney coverage. Since in the setting of the German Diabetes Study (GDS)^10^, both MR image sequences were acquired for a large group of volunteers, this gives us the opportunity to compare the outcomes of MRI protocols with different kidney coverage. In this view, determining how estimates of RSF obtained from suboptimal whole-body MRI protocols are compared to high-resolution MR images can be important in the future for retrospective analysis RSF in a larger cohort. This information can be important for pooling existing image datasets from cohorts that were scanned using different MRI protocols.

Quantifying RSF volume throughout the entire kidney along with other kidney structures would improve the accuracy of the analysis. Meanwhile manual delineation of small and ill-defined kidney structures is very labor-intensive and time-consuming, especially when the whole kidney is covered with contiguous slices. In this context, a fully automated method using current advanced image analysis approaches, particularly U-shaped convolutional neural networks (U-Net), would be highly useful for volumetric analysis of kidney structures. With their encoder-decoder architecture, U-Nets are designed to capture both local and global contextual features, enabling efficient discrimination between RSF and renal parenchyma (RP), which lie in close proximity and often differ only by subtle MRI intensity contrasts. In addition, this approach could reduce the inter-reader variability. Deep learning-based segmentation methods have already promise in detecting RP and small tumors^11^. However, only few studies applied similar automated or semi-automated techniques to the detection and quantification of small renal compartments^12,13^.

In the present study we evaluated the performance of dedicated U-Net models optimized on a nnU-Net framework^14^, a well-established benchmark for automated biomedical image segmentation, to accurately segment and quantify the kidney structures within the setting of the GDS. Differences in the estimation of RSF and RP volumes using either a single slice or a volumetric approach were evaluated across people with different glycaemic status. Automatic segmentation and quantification of kidney structures was deployed to evaluate anatomical differences between male and female and across individuals with and without diabetes. Finally, we assessed the accuracy of whole-body MR image protocols acquired with partial tissue coverage as compared to high-resolution MRI enabling the precise quantification of RSF across the whole kidney.

## Results

### Performance of U-Net models for the segmentation of RSF and RP volumes

**Fig. 1:**
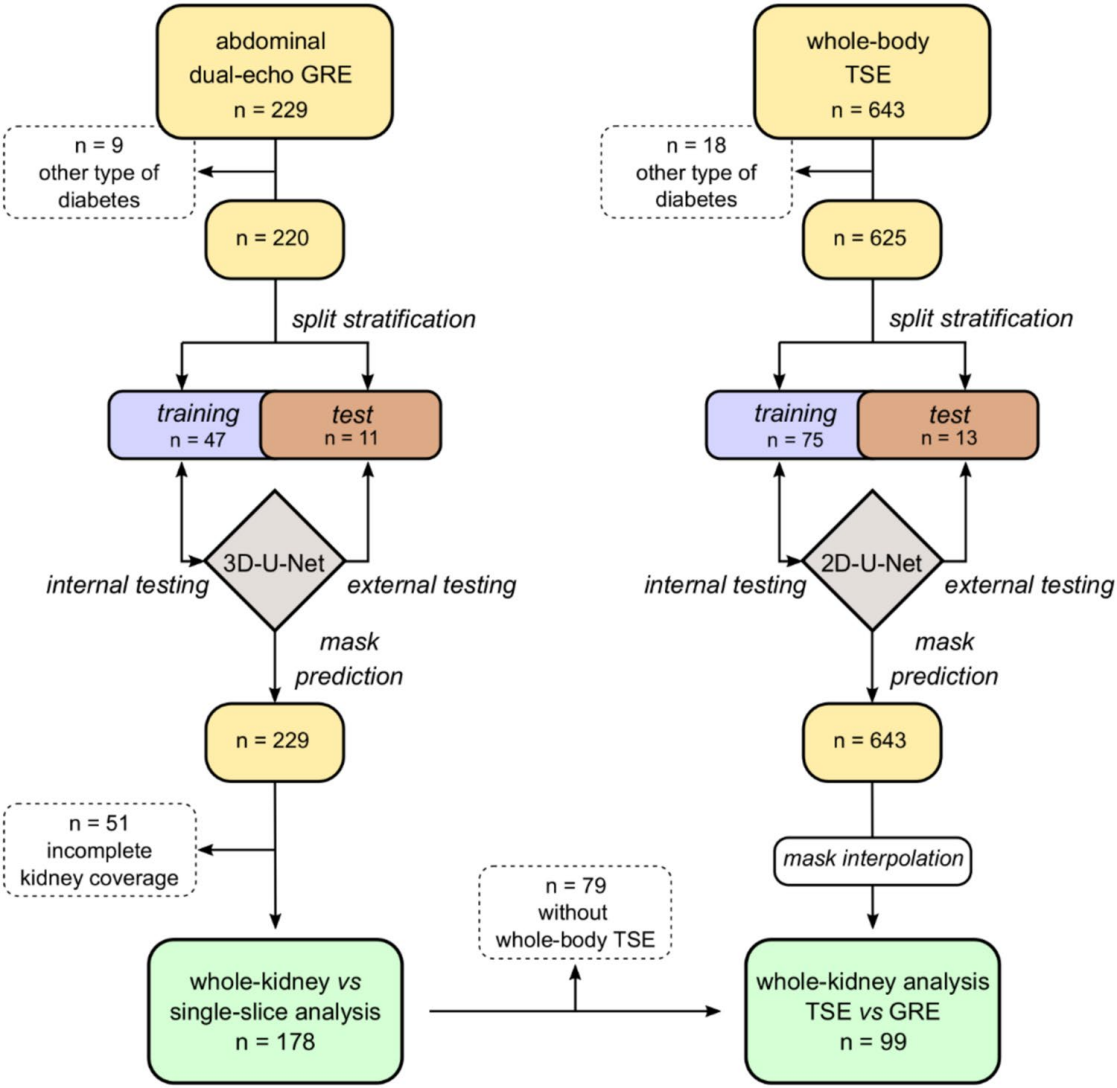
Flowchart for training and testing of U-Net models and downstream analysis. Schematic representation of the partitioned datasets used in the study, including abdominal dual-echo gradient echo (GRE) and whole-body turbo-spin echo (TSE) images that were used for the training of 3D- and 2D-U-Net models, respectively. For the analysis, 178 individual datasets were selected to compare the quantification of kidney structures using either whole-kidney or single-slice approach. A total of 99 individuals that underwent both imaging protocols were used for the comparison of two imaging protocols.

The performance of the 3D-U-Net model trained on water- and fat-reconstructed GRE images was evaluated internally on the test set (Fig. 1 and Table 1). The segmentation of the model yielded high DSC values equal to 0.96 ± 0.01 and 0.81 ± 0.05 for RP and RSF volumes, respectively, on the test set (Table 2). Similar tendencies are reflected by the other metric, with the RSF volume showing slightly worse outcomes.

**Table 1:** Demographic information of each partitioned dataset.

	abdominal dual-echo GRE			whole-body TSE		
	all (n = 220)	training (n = 47)	test (n = 12)	all (n = 625)	training (n = 75)	test (n = 13)
Age (y)	51.3 ± 13.7	51.9 ± 13.0	56.8 ± 12.3	46.2 ± 13.6	47.5 ± 13.7	48.2 ± 13.6
Sex (female %)	73 (33.0)	15 (31.3)	5 (41.7)	213 (34.1)	34 (44.6)	7 (53.8)
BMI (kg/m^2^)	27.1 ± 4.4	26.6 ± 4.2	28.8 ± 5.8	27.3 ± 5.0	27.7 ± 5.1	27.4 ± 4.3
Glycaemic status:						
Control: n° (%)	56 (25.3)	16 (33.3)	3 (25.0)	167 (26.7)	25 (32.4)	1 (7.7)
T1D: n° (%)	84 (38.0)	14 (29.2)	4 (33.3)	242 (38.7)	17 (23.0)	6 (46.2)
T2D: n° (%)	81 (36.6)	18 (37.5)	5 (41.7)	216 (34.6)	33 (44.6)	6 (46.2)

Values are given as mean ± standard deviation (SD) or number of patients with percentage in brackets BMI: Body Mass Index GRE: Gradient-Echo T1D: Type 1 Diabetes T2D: Type 2 Diabetes TSE: Turbo-Spin Echo.

**Table 2:** Metrics of U-Net model performance for whole-kidney segmentation against manual segmentation.

		DSC	IoU	Accuracy	Sensitivity	Precision	Specificity
abdominal dual-echo GRE (n = 12)	RP	0.96 ± 0.01	0.92 ± 0.02	1.00 ± 0.00	0.96 ± 0.02	0.95 ± 0.01	1.00 ± 0.00
	RSF	0.81 ± 0.05	0.68 ± 0.06	1.00 ± 0.00	0.79 ± 0.07	0.83 ± 0.08	1.00 ± 0.00
whole-body TSE (n = 13)	RP	0.95 ± 0.01	0.90 ± 0.01	1.00 ± 0.00	0.94 ± 0.02	0.96 ± 0.01	1.00 ± 0.00
	RSF	0.80 ± 0.02	0.68 ± 0.03	1.00 ± 0.00	0.77 ± 0.08	0.86 ± 0.06	1.00 ± 0.00

Values are given as mean ± standard deviation (SD) GRE: Gradient-Echo DSC: Dice Similarity Coefficient RP: Renal Parenchyma RSF: Renal Sinus Fat TSE: Turbo-Spin Echo.

**Fig. 2:**
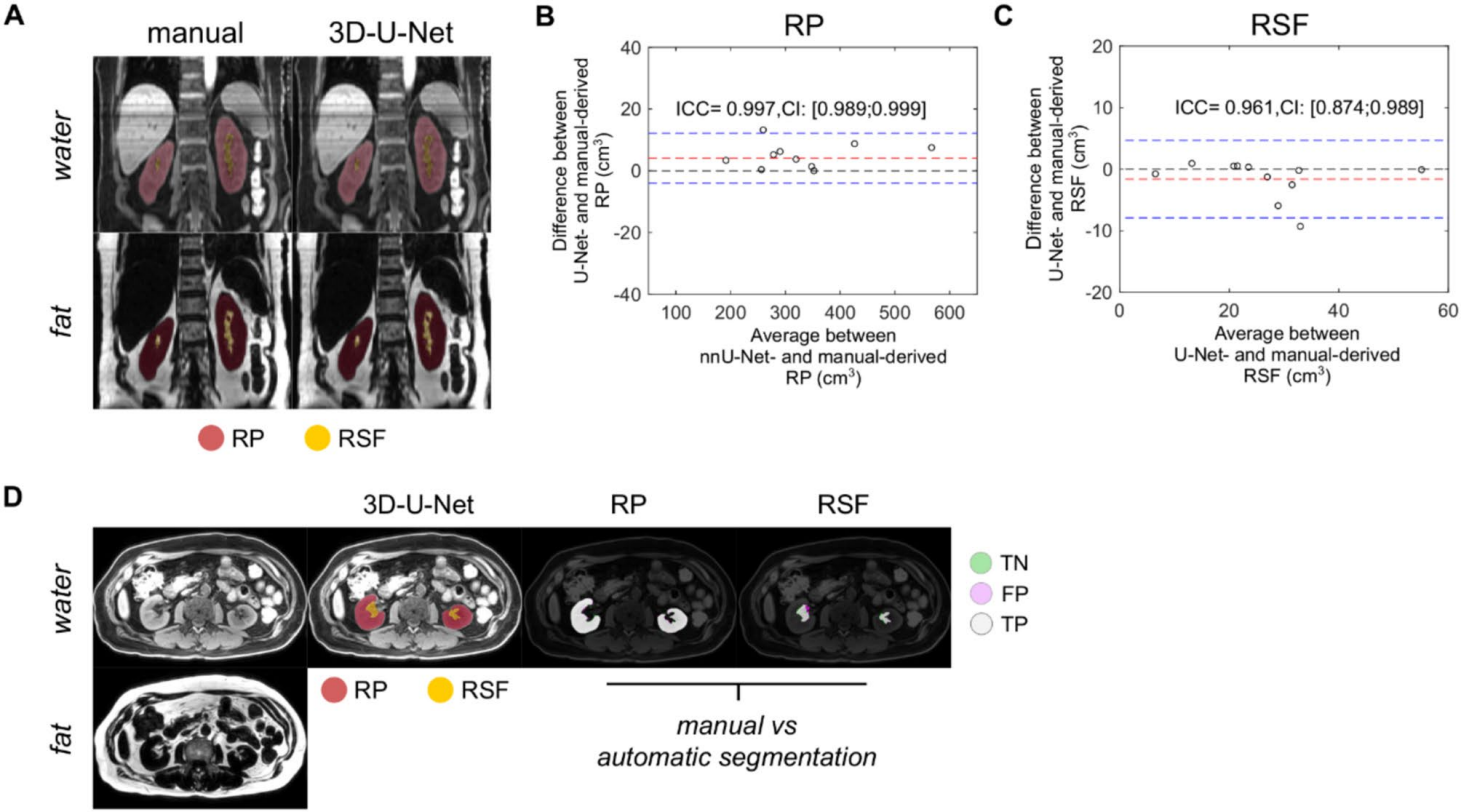
Accuracy of U-Net for the segmentation of renal sinus fat from abdominal GRE images. (**A**) Representative coronal projection view of water- and fat-reconstructed images derived from the dual-echo GRE images are displayed with the respective masks overlaid for renal parenchyma (RP) and renal sinus fat (RSF), which were derived manual or using the trained 3D-U-Net model. Bland-Altman plots and intraclass correlation coefficients (ICC) are shown to determine the agreement (**B**) RP and of (**C**) RSF volume (cm^3^) from the test set. The mean differences (red dashed line) and limits of agreement (blue dashed line) are shown for both tests. (**D**) Segmentation of RP and RSF volume from water- and fat-reconstructed axial images and comparison with manual segmentation for RP and RSF volume with each voxel labelled as true negative (TN, green), false positive (FP, purple) or true positive (TP, grey).

In line, the estimates obtained from the segmentation of RP and RSF volumes were in good agreement with the standard reference, showing mean differences of 2.2 cm^3^ [LoA: -13.46; 17.89] cm^3^, ICC of 0.997, [95% CI: 0.989; 0.999] for the quantification of RP volume and -1.30 cm^3^, LoA: [-7.68; 5.12] cm^3^, ICC of 0.961, [95% CI: 0.874; 0.989] for the RSF volume (Fig. 2a-c). An illustrative example showing the spatial accuracy of the model’s predictions for RP and RSF compartment detection on a test set is shown in Fig. 2d and Supplementary Information Fig. 1.

**Fig. 3:**
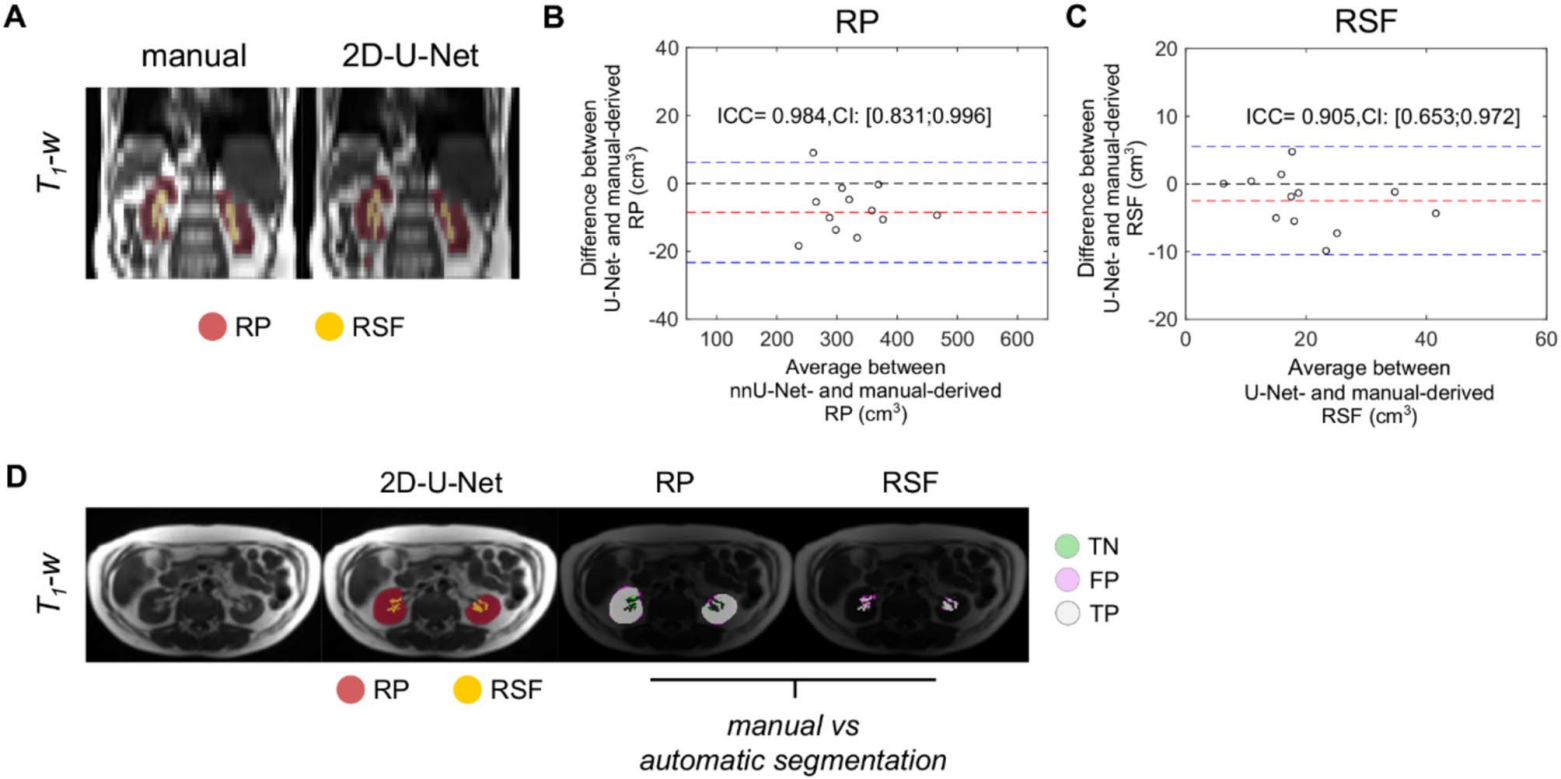
Accuracy of U-Net for the segmentation of renal sinus fat from whole-body TSE images. (**A**) A representative coronal projection view of TSE images with the respective overlaid masks produced either from the manual analysis or by the trained 2D-U-Net model. Bland-Altman plots and intraclass correlation coefficients (ICC) are shown for the quantification of (**B**) RP and (**C**) RSF volume (cm^3^) on the test set. Mean differences (red dashed line) and limits of agreement (blue dashed line) are displayed. (**D**) Masks of RP and RSF volume derived from the original axial images along with the comparison with manual segmentation showing voxels labelled as true negative (TN, green), false positive (FP, purple) or true positive (TP, grey).

The segmentation of RSF volume derived by the 2D-U-Net model trained with whole-body TSE images yielded DSC equal to 0.95 ± 0.01 and 0.80 ± 0.02 for RP and RSF volume, respectively, in the hold-out test set (Table 2). Similarly as shown with the previous model, the quantification of RP and RSF volumes was in well agreement with the reference standard, with ICC values equal to 0.997 [95% CI: 0.987; 0.999] and 0.905 [95% CI: 0.653; 0.972] for RP and RSF volume, respectively (Fig. 3a-c). The estimates of RSF produced by manual segmentation were slightly higher values compared to the RSF volumes derived by the model, with a mean difference of -2.48 cm^3^ [LoA: -10.47; 5.51]. The accuracy of the predictions for RP and RSF compartments on a representative dataset from the test set is depicted in Fig. 3d and Supplementary Information Fig. 2.

### Assessment of RP and RSF by whole-kidney and single-slice analysis

**Fig. 4:**
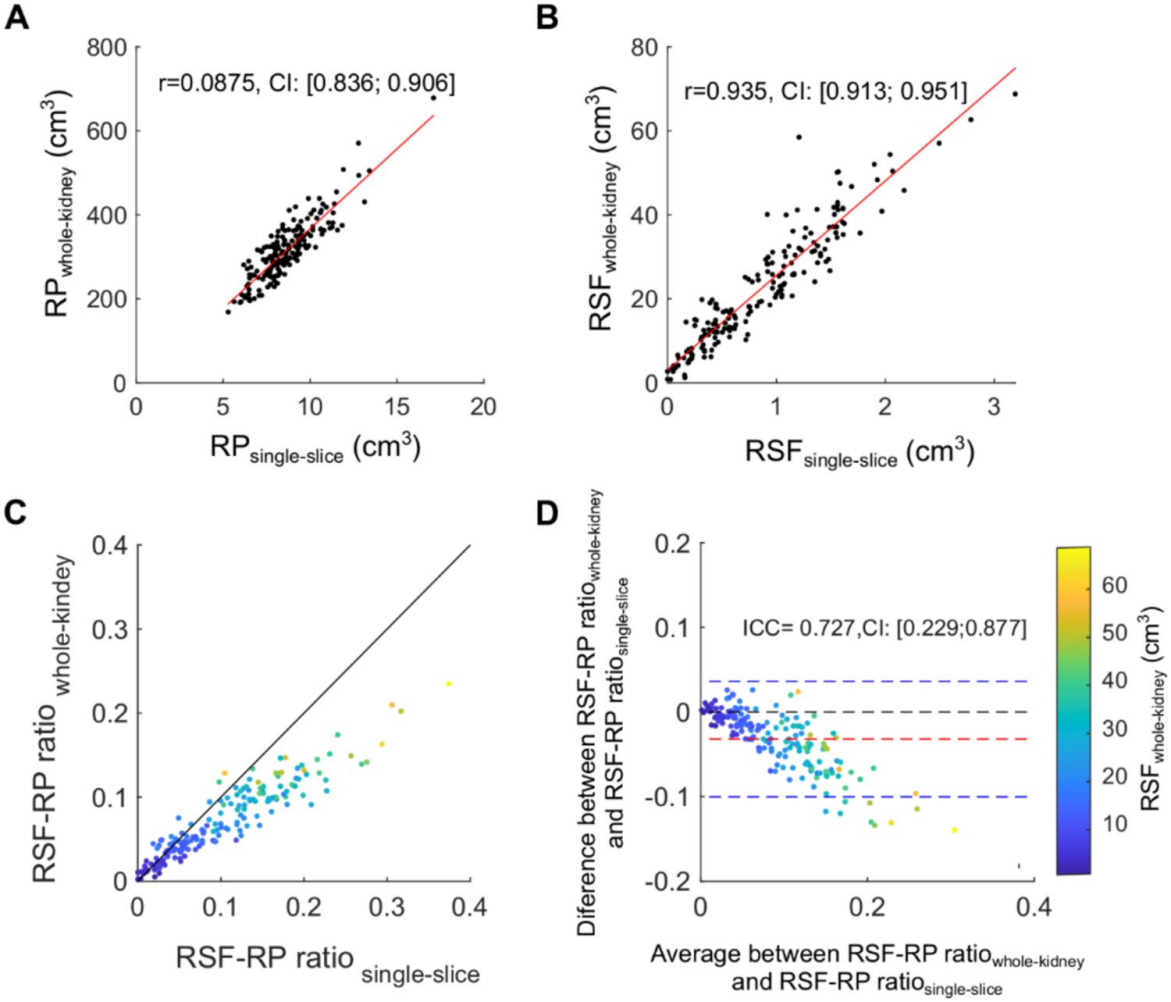
Comparison between whole-kidney and single-slice analysis. The scatter plots display the estimates for (**A**) renal parenchyma (RP), (**B**) renal sinus fat volume (cm^3^) and (**C**) RSF-RP ratio calculated using either the central slice (single-slice) or all the slices (whole-kidney). Linear regression (red line) and Pearson’s *r* coefficients with 95% confidence intervals (CI) are displayed. (**D**) Bland-Altman plots and intraclass correlation coefficient (ICC) for RSF-RP ratio calculated between the two analytical methods, with mean difference (red dashed line) and limits of agreement (blue dashed line). Data points are colour labelled using the RSF volume (cm^3^) calculated using all the slices of the kidneys.

A further comparison was performed to assess differences in estimating the renal compartment volumes using high-resolution dual-echo GRE images using either a single-slice or whole-kidney analysis. From the correlation analysis, we observed that the estimates of RP and RSF volumes correlated well between the two approaches, with Pearson’s *r* coefficients equal to 0.875 [CI: 0.836; 0.906] and 0.935 [CI: 0.913; 0.951], respectively (Fig. 4a-c). However, the resulting RSF-RP ratio showed only a moderate level of agreement between the two methods, with ICC = 0.727 [CI: 0.229; 0.877] (Fig. 4d). Differences between single-slice and whole-kidney analysis were negligible at low values, while they were progressively larger at a higher magnitude RSF-RP ratio and RSF volume measured from the whole-kidney, with a mean difference in the RSF-RP ratio of -0.03 [LoA: -0.10; 0.04] in the RSF-RP ratio. In general, the RSF-RP ratios calculated from a single-slice approach produced greater estimates compared to those calculated from all the slices (whole-kidney), with the largest percentage difference in the group of individuals with T2D showing higher RSF-RP ratio (Table 3 and Supplementary Information Fig. 3).

**Table 3:** Whole-kidney vs single-slice analysis of abdominal dual-echo GRE images across control, T1D and T2D.

	RSF-RP ratio (n = 178)		
	Control (n = 45)	T1D (n = 72)	T2D (n = 61)
Single-slice	0.10 ± 0.07	0.08 ± 0.06	0.13 ± 0.08
Whole-kidney	0.07 ± 0.04	0.06 ± 0.04	0.09 ± 0.05
[Δ] (%)	-38.3 ± 44.6	-31.0 ± 43.9	-47.0 ± 51.3

Mean ± standard deviation (SD) of the RSF-RP ratio calculated by from one central slice (single-slice) or using the entire volume of the kidney (whole-kidney) GRE: Gradient-Echo RP: Renal Parenchyma RSF: Renal Sinus Fat T1D: Type 1 Diabetes T2D: Type 2 Diabetes.

### Comparison of RP and RSF across male, female and persons with different glycaemic status

The statistical comparison of kidney structures showed that RSF volume differed significantly across gender (*F*(1) = 7.17, *p* = 0.0081) and glycaemic status (*F*(2) = 8.37, *p* = 0.0003). More specifically, female displayed 5.53 cm^3^ [95% CI: 1.45; 9.61] lower in the RSF content as compared to men. People with T1D showed the lowest content of RSF, showing a mean difference of -9.38 cm^3^ [95% CI: -14.93; -3.82] (*p* = 0.0002) in comparison with people with T2D, and -3.65 cm^3^ [95% CI: 2.43; -9.72] (*p* = 0.4466) with heathy controls, respectively. By contrast, no significant differences were found in the RP volume across glycaemic status (*F*(2) = 0.8, *p* = 0.4528). Further, the RSF-RP ratio was significantly different across glycaemic status (*F*(2) = 7.35, *p* = 0.0009), while no differences were found across gender (*F*(1) = 1.87, *p* = 0.173).

Regarding differences in the volume between the left and right kidney structures, the RSF volume of the left kidney was 20–40% larger than the right kidney across all the groups (Supplementary Information Fig. 4a, b and Supplementary Information Table 2). A slight difference was found in the RP volume of the left kidney showing values between 2–6% than the right kidney.

### Comparison between whole-body TSE and abdominal GRE images in quantifying the RP, RSF and RSF/RP ratio

**Fig. 5:**
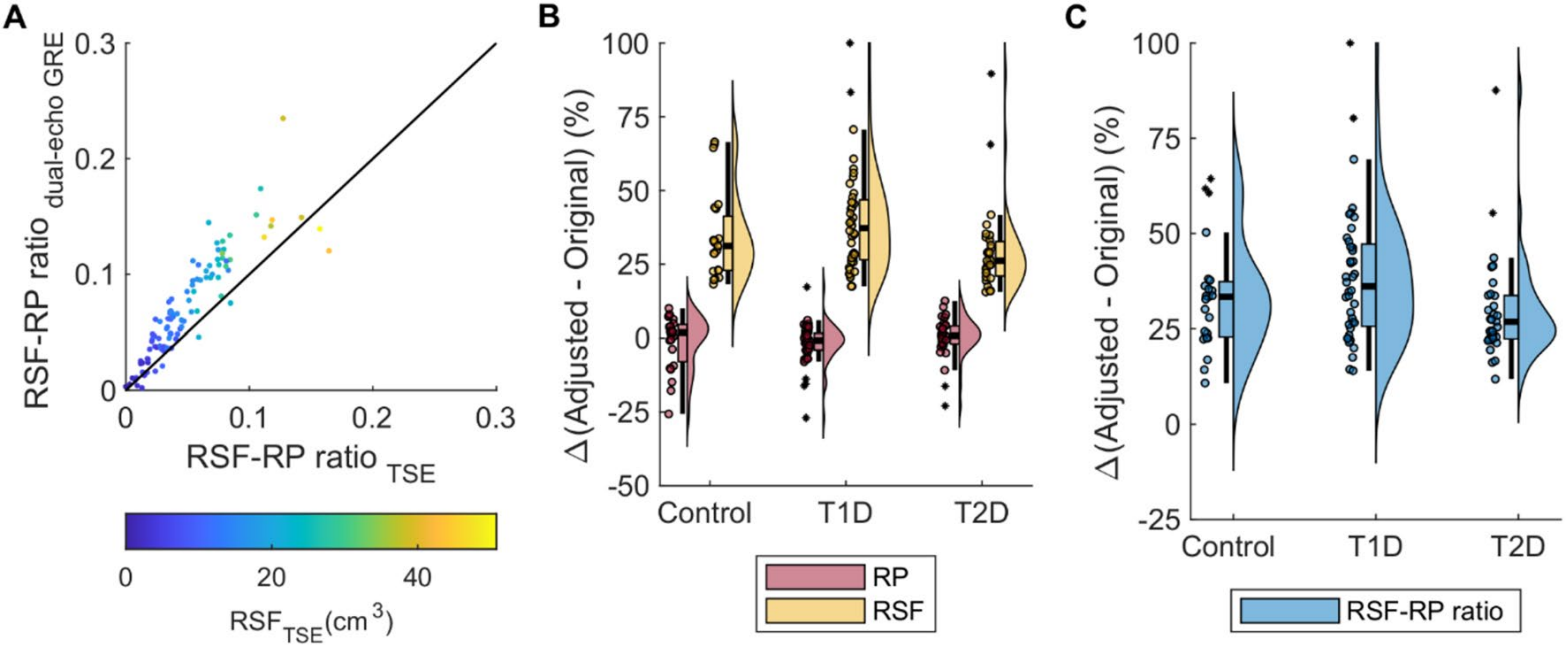
Comparison of GRE and TSE images with different kidney coverage: estimates of kidney RSF and RP across persons with different glycaemic states. (**A**) The scatter plot displays the estimates of renal sinus fat-to renal parenchyma ratio (RSF-RP ratio) calculated from the whole-body turbo spin-echo (TSE) acquired and abdominal dual-echo gradient echo (GRE) images. Linear regression (red line) and identity lines (black line) are displayed. The half-violin plots and the corresponding scatter and boxplots display the percentage difference between the original values and the adjusted values for (**B**) RP, RSF volume and (**C**) the RSF-RP ratio (%) obtained from the whole-body TSE images.

To evaluate the agreement between whole-body TSE imaging and abdominal dual-echo GRE images, we investigated differences in the outcome of RP and RSF from individuals who underwent both imaging protocols. Linear regression analysis indicated a good agreement in the assessment of RP volume between the two imaging protocols (Supplementary Information Fig. 5a). Differences between the two MRI protocols were more pronounced for the estimation of RSF volume (Supplementary Information Fig. 5b). Further, these differences led to a discrepancy of the RSF-RP ratio between the two imaging protocols, with the discrepancy becoming more pronounced at higher RSF volume (Fig 5a).

When compared with their respective adjusted values the original values for RSF as well as their RP-RSF ratio were 30–40% lower across all the groups (Fig 5b, c and Table 4). By contrast, only small differences were observed by comparing the RP volume before and after adjustment.

**Table 4:** Quantification of RP, RSF and RSF-to-RP ratio from whole-body TSE images, before and after adjustment.

	Control (n = 23)			T1D (n = 40)			T2D (n = 31)		
	RP (cm^3^)	RSF (cm^3^)	RSF-RP ratio	RP (cm^3^)	RSF (cm^3^)	RSF-RP ratio	RP (cm^3^)	RSF (cm^3^)	RSF-RP ratio
Original values	319.0 ± 89.8	15.1 ± 9.1	0.048 ± 0.032	306.4 ± 85.5	12.5 ± 9.3	0.042 ± 0.032	331.9 ± 79.4	20.9 ± 12.7	0.063 ± 0.039
Adjusted values	317.7 ± 65.8	21.2 ± 9.8	0.067 ± 0.029	308.4 ± 62.6	18.4 ± 10.1	0.061 ± 0.034	327.1 ± 58.2	27.6 ± 13.7	0.084 ± 0.042
[Δ] (%)	1.3 ± 9.2	34.4 ± 14.5	33.4 ± 14.4	1.9 ± 7.2	39.7 ± 17.7	38.7 ± 17.8	0.3 ± 7.1	29.3 ± 14.9	29.9 ± 14.0

Values are given as mean ± standard deviation (SD) in control, T1D and T2D GRE: Gradient-Echo RP: Renal Parenchyma RSF: Renal Sinus Fat T1D: Type 1 Diabetes T2D: Type 2 Diabetes TSE: Turbo-Spin Echo.

## Discussion

In the present study, the performance of trained U-Net models to quantify the RP and RSF compartments was assessed. The dedicated U-Net models produced accurate segmentation and quantification of RP and RSF with a high degree of agreement with manual segmentation. In particular, the automatic assessment of the whole-kidney showed agreement levels with manual annotation comparable to inter-readers agreement reported in a previous study of CT scans, where only a single scan was evaluated^15^. For both MRI protocols under investigation, a larger discrepancy between manual and automatic segmentation was observed for the delineation of RSF. This is likely due to the sensitivity of DSC for the assessment small regions^16^ as well as by the difficulty to manually delineate the borders of such small organ substructures. Similar biases for small organ substructures have been shown in a recent study where a U-Net model was used to identify different renal structures^12^. Nevertheless, in line with previous studies, our results demonstrated that our models generalize well on unseen datasets for accurately segmenting RP and RSF compartments.

Our study aimed to investigate the importance of using whole-kidney analysis as compared to a single-slice approach as reference to assess the kidney structures. In line with a previous report on CT scans^15^, the two methods correlated well for the estimation of RSF and RP content. However, we found a clear tendency using a single-slice approach to overestimate the RSF-RP ratio. Interestingly, we found increased bias in individuals with elevated RSF volume and T2D. This can be explained by the lower proportion of renal parenchyma at the level of the renal pelvis and the heterogeneity in the distribution of fat depots throughout the kidney^17^. Taken together, our results indicate that whole-kidney analysis is more advisable than a single-slice approach when linking RSF-RP ratio to the risk to develop nephropathy in individuals with T2D^7^.

Interestingly, whole-kidney analysis allowed us to discriminate anatomical differences in the renal compartments between male and female as well as between the left and right kidneys (see Supplementary Information Document). Regarding this, the renal parenchyma of the left kidney was slightly larger than the contralateral kidney, whereas we found an accumulation of renal sinus fat in the left kidney between 20 and 40% higher than in the right kidney. Those findings are not novel but highlight known anatomical differences between left and right kidneys and corroborate previous studies showing similar results regarding differences in kidney size between the genders and the higher accumulation of fat in the left kidney^12,17–19^.

People with T2D analysed using a volumetric approach displayed larger renal sinus fat volume, which is known to be associated with hypertension and CKD^3,6^. Although the association of obesity with hypertension and CKD is well established, the pathophysiological mechanisms leading to those conditions are not yet well understood. It has been hypothesized that RSF, which represents triglyceride accumulation in adipocytes in the hilus of the kidneys, could impair renal function via the compression of the renal artery or by other mechanisms. However, whether the accumulation of renal sinus fat indeed contributes to the onset of CKD requires future studies^3,8^. The differences in RSF content across persons with normal glucose regulation, T1D and T2D should be further investigated to clarify the role of this fat compartment in the etiology of CKD, however this is beyond the scope of the current work.

Automatic segmentation of the renal compartments is a prerequisite for downstream analysis of imaging biomarkers, including the extraction of morphological features by using a radiomic approaches, as well as for evaluating fat fraction indexes within the renal parenchyma, which has been recently shown to contribute to the progressive loss of renal function in people with T2D^20,21^.

Further, we investigated the accuracy of a whole-body TSE images acquired with partial tissue coverage (with interslice-gaps) using as reference abdominal GRE images with high spatial resolution and full-kidney coverage. Even tough interpolation of volume was used to compensate for interslice-gap, the RSF was consistently lower using a whole-body MRI protocol as compared to reference values. Those results are not surprising, however highlight the difficulty in the detection of fat depots that are unevenly distributed across the renal sinus using protocols with interslice gaps. This may lead to an underestimation in the absolute quantification of RSF volume. Nevertheless, we showed that by correlating the RSF and RP outcomes from different MRI protocols, it is possible to correct for this bias and thereby derive more accurate RSF-RP ratio. Indeed, after such correction, values of RSF obtained from people with normal glucose regulation and people with different glycaemic status are in better agreement with the ranges reported in literature^7^. Therefore, our results indicate that differences in image analysis methods and kidney coverage should be carefully evaluated when comparing small organ substructures such as the renal sinus fat from different studies. In this regard, assessing the agreement of different MRI protocols and imaging modalities by leveraging deep neural networks may be a viable approach to pool image data resources from multiple centers.

Our models performed very well on both the respective test sets, although further intra- and inter-observer studies would be insightful to better determine the precision of such models compared to human performance and on external datasets from different sites and scanner vendors. Further, repeated rounds from multiple readers and refinement of regions based on a common consensus will improve the quality insurance of manual references for the segmentation of renal sinus fat^22^. Importantly, the models were trained and tested on a population that meets the inclusion criteria of the GDS, which may limit their applicability to individuals with other kidney diseases or tumors.

Our study focuses on the evaluation of dedicated nnU-Net models, each separately trained on different MRI sequences to segment kidney structures. A systematic comparison with additional state-of-the-art segmentation methods was beyond the scope of this study. Nevertheless, models trained on the nnU-Net-based models remains a strong benchmark and showed already promising results in segmenting different anatomical structures from MR images in large population cohorts [23,24]. Future work should also investigate how more advanced architectures perform relative to the nnU-Net models evaluated here. This includes transformer-based networks as well as convolutional neural networks enhanced with attention mechanisms [25,26]. This study utilizes MR image data acquired from the same individuals on a single MR scanner, an approach that ensures direct comparison but can be challenging to implement in multi-center studies.

Validation of biomarkers obtained from different MRI protocols and multiple centers would benefit from the implementation of recent foundation models in biomedical image analysis, which can be used to obtain anatomical information across various organs, imaging modalities (MRI, CT, etc..), MRI protocols, field strengths and vendors [27].

In summary, the deployment of dedicated deep neural networks provides an efficient and accurate for segmenting and obtaining RP and RSF volumes. Our findings show that single-slice analysis of RSF from high-resolution MRI is influenced by kidney size and tends to consistently overestimate the content of RSF, particularly at higher RSF volumes. Additionally, we established a correction factor based on high-resolution MRI to produce more accurate estimates of renal sinus fat from a whole-body MRI protocol with partial kidney coverage. Ultimately, whole-kidney automated segmentation enables reliable measurements of RP and RSF volume, offering a valuable tool for advancing our understanding of CKD progression in individuals with diabetes and metabolic disorders across large cohorts.

## Methods

### Study population

This retrospective study was conducted within the setting the German Diabetes Study (GDS) [28] for the evaluation of the performance of dedicated deep neural networks trained on different MRI protocols to determine the accumulation of renal sinus fat. People with normal glucose tolerance and individuals with type 1 (T1D) and type 2 diabetes (T2D) were included. The GDS follows people with recent-onset T1D and T2D, aged 18–69 years, for comprehensive metabolic phenotyping and assessment of diabetes-related complications and comorbidities in regular visits every 5 years. The clinical study is conducted according to the Declaration of Helsinki (2013 version) and approved by the ethics committee of the Medical Faculty of the Heinrich Heine University, Düsseldorf (reference number 4508) and registered at Clinicaltrials.gov (identified number: NCT01055093). All participants gave written informed consent. For the current study, only participants who underwent MR examinations were included.

### In vivo MRI measurements

All MRI measurements were performed on a 3 Tesla clinical MR scanner (Achieva X-series, Philips Healthcare, Best, The Netherlands). The general parameters of the respective MRI protocols are summarized in the Supplementary Information Table 1.

The whole-body MRI protocol was carried out in prone positioning by sequentially acquiring multiple stacks of 2D turbo-spin-echo (TSE) T1w images to cover the entire body length. During the same imaging session, abdominal MRI measurements were carried out in supine positioning by acquiring an unenhanced and transversal dual-echo 3D gradient-echo (GRE). The abdominal images were planned in order to cover the whole liver, and the acquisition was performed within a single breath-hold. Water, fat, in-phase and opposed-phase images were reconstructed.

### Training of U-Net models

All demographic information from the partitioned data are displayed in Table 1.

For GRE dataset, participants with other type of diabetes (n = 9), including Maturity Onset Diabetes of the Young (MODY) and Latent Autoimmune Diabetes in Adults (LADA), were excluded for the training and evaluation of the model (Fig. 1). The training (n = 47) and the test sets (n = 12) were obtained by random selection of datasets which were previously stratified based on different ranges of body mass index (BMI) (15 < BMI < 25; 25 < BMI < 28; 28 < BMI < 50) and sex. The reference standards were produced by a single independent trained operator (RK) on SliceOmatic® (Tomovision, Magog, Canada). Manual delineation of the RP and RSF compartments was performed on every fourth slice using the fat-reconstructed images as reference source image. Then, interpolation of masks was performed to obtain the manual annotations across all the slices.

For the TSE dataset, 625 image datasets were stratified based on BMI and sex as previously described for the GRE datasets to randomly select the training (n = 75) and test sets (n = 13). The reference standards for the RP and RSF compartments were drawn on all available slices by a separated independent operator (KM).

The U-Net architectures were optimized using a no-new (nn) U-Net framework^14^ for both the dual-echo GRE (patch size = 224 × 192 × 56, batch size = 2, epochs = 1000) and the TSE image datasets (patch size = 256 × 256, batch size = 49, epochs = 1000). For the GRE images, both the water- and fat-reconstructed images were used for the training of the model. Further details of the 3D-U-Net and 2D-U-Net architectures including number of layers, feature maps and block of operation applied are reported in Supplementary Information Fig. 6. Pre-processing of the input images included scaling of signal in the range [0.01–1] and noise reduction using a normalized average filter. We used a combination of Gaussian Mixture model and connect component analysis to segment the background and foreground from the GRE and TSE images. Thus, the background, foreground, renal parenchyma and renal sinus fat were fed to the 3D-U-Net and to the 2D-U-Net as reference standard (Supplementary Information Fig. 7). All the images and corresponding reference manually drawn masks of the training sets were visually inspected by a second reader before the training of the networks.

The training was performed using a fivefold cross-validation on dedicated workstation on a GPU NVIDIA RTX3090. The training scheme included stochastic gradient descent with a high initial learning rate (0.01) and a large nesterov momentum (0.99) used as optimizer. A decay-learning rate was used, decreasing almost linearly to 0 over the 1000 epochs. Data augmentation was implemented on the fly, including rotations, scaling, Gaussian noise, Gaussian blur, brightness, contrast, simulation of low resolution, gamma correction and mirroring. More details on the optimization of model architecture was described previously^14^.

### Evaluation of models for the segmentation of RP and RSF volumes

The prediction of RP and RSF masks was performed using the trained 3D-U-Net and 2D-U-Net models for GRE and TSE images, respectively, using an ensemble configuration and, thus, by averaging the softmax probabilities of the 5 models, which trained during cross-validation. The performance of models for segmenting the RP and RSF was evaluated on the respective test sets (Fig. 1). To this end, the trained models by computing the dice similarity coefficient (DSC), intersection over union (IoU), accuracy, sensitivity, precision and specificity using as reference standard the manual annotations previously described.

Following the generation of masks using the respective ensemble 3D-U-Net and 2D-Net models (Supplementary Information Fig. 6), post-processing of manual and U-Net-derived masks included connect component analysis to exclude the presence of unspecific regions.

### Assessment of renal parenchyma and renal sinus fat and comparison of whole-kidney and single-slice analysis

For the quantification, the masks for RP and RSF were generated using the trained ensemble 3D-U-Net model across the entire GRE dataset (n = 229), including both training and test sets, following post-processing to exclude the unspecific regions as described previously.

For quality insurance, the abdominal GRE images (n = 229) were visually inspected for containing the whole kidney volume and leading to the selection of 178 individual datasets. Those image datasets that did not cover the caudal part of the kidney (n = 51) were excluded from the analysis. All datasets were visually inspected for correct prediction of RP and RSF volume in the region of interest. The volumes for RP and RSF were calculated by the sum of the respective regions across all slices. The ratio of renal sinus fat-to-renal parenchyma (RSF-RP ratio) was derived by dividing the RSF volume over the RP volume.

The assessment of RP and RSF using a single-slice approach was performed using the same masks generated from the trained 3D-U-Net model. The estimates for RP and RSF were calculated by summing the largest cross-sectional areas of the left and the right kidneys. Differences between the RSF-RP ratio were evaluated across the groups, including healthy individuals (n = 45) and people with T1D (n = 72) and T2D diabetes (n = 61).

### Comparison of MRI sequences with different kidney coverage

The accuracy of whole-body TSE images for the quantification of RP and RSF volumes was assessed using as reference the abdominal dual-echo GRE images acquired with full kidney coverage.

From the previous 178 datasets selected to include the entire kidney volume, the dataset was further reduced to 99 image datasets by excluding those individuals (n = 79) that did not undergo whole-body TSE imaging acquisition (Fig. 1).

For the quantification, the masks for RP and RSF were firstly derived from the respective trained 2D- and 3D-U-Net models and the volumes of RP and RSF were calculated using the sum of the respective left and right kidney. As previously, all predicted masks for RP and RSF volume segmentation were visually inspected. For the whole-body TSE images acquired with partial kidney coverage, we interpolated RP and RSF volumes across all the slices containing the kidney to compensate for the interslice-gaps.

Differences between the original values of RP, RSF volumes, before and after adjustment were evaluated in healthy individuals (n = 23) and people with T1D (n = 40) and T2D diabetes (n = 31). People with other type of diabetes (n = 5) were excluded from this analysis. To this end, the original values were adjusted using the linear regression coefficients of models for RP and RSF volume (see Supplementary Information Fig. 5), respectively, reflecting the relationship between TSE and GRE images.

### Statistics

Agreements between RSF and RP volumes obtained by manual- and U-Net-derived segmentations were assessed by using the Bland–Altman plots, including the calculation of the mean differences and limits of agreement (LoA). The intraclass correlation coefficients (ICC) and 95% confidence intervals (CI) were computed to determine the levels of agreement between automatic and manual image analysis for the quantification of kidney structures from the whole-kidney. To determine the agreement between whole-kidney and single-slice analysis the Pearson’s *r* coefficients was calculated to assess RP and RSF volume from GRE images (n = 178) displaying full coverage of the kidneys. Bland–Altman plots and ICCs were computed to determine the differences between the two method in the assessment of RSF-RP ratio. The relationships between whole-body TSE or abdominal dual-echo GRE images in the assessment of RP and RSF volume were determined by linear regression analysis. Statistical comparison by two-way ANOVA and Bonferroni post-hoc analysis was performed to assess differences in the RSF and RP volumes across people with different gender and glycaemic status (control, T1D and T2D). For this analysis, differences were evaluated using the sum of the volumes from both the right and the left kidneys. Further, differences between the left and right kidney were evaluated by computing the mean difference across people with different gender and glycaemic status.

## Supplementary Information

Supplementary Information.

## Data Availability

Data of this study can be made available from the corresponding author upon request and subject to institutional approval.
