# Supplementary material for: A comparison study to assess U-Net driven volumetric versus single-slice analysis and MRI sequences with different volume coverage to detect renal sinus fat in people with and without diabetes
Source: Sci Rep. 2026 Feb 3;16:4826. doi: 10.1038/s41598-025-33098-0 (PMC12873195; doi:10.1038/s41598-025-33098-0)
Supplement: Supplementary file 1 — Supplementary Information. [file 41598_2025_33098_MOESM1_ESM.docx]

# Supplementary Information Documents

## Analysis of RSF and RP between left and right kidney

Anatomical differences between left and right kidney were evaluated on dual-echo GRE images displaying full-kidney coverage (n=187). As compared to the right kidney, the compartments of the left kidney were larger across all the groups, with values up to 7% and 36% in average higher in the RP and RSF compartments, respectively, (Supplemental Information Fig. 2a-c and Table 2).

# Supplementary Information Figures


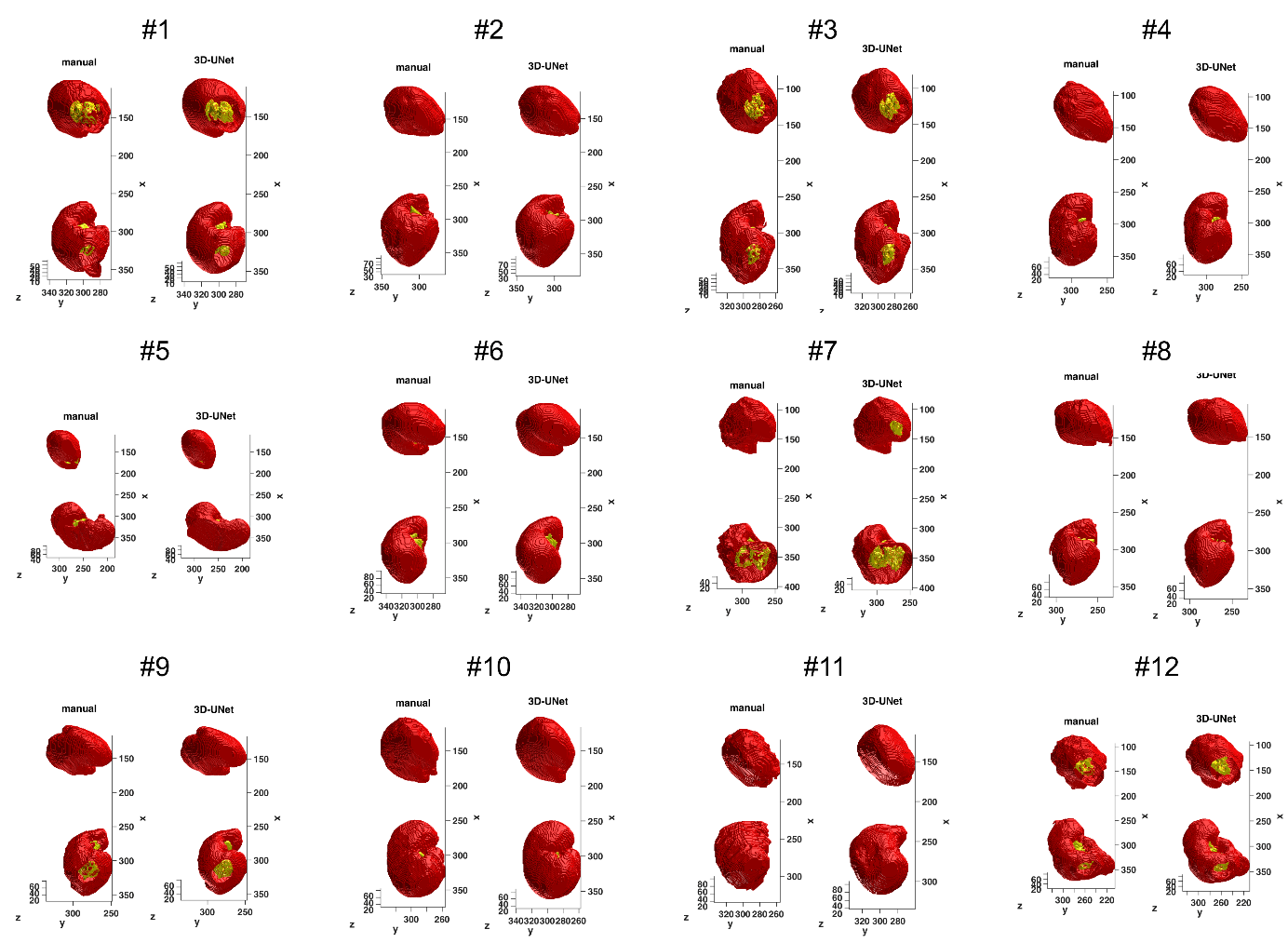


Fig. 1: Illustration of manual and automatic segmentation from high-resolution abdominal MRI

Display of 3D surface representation of the anatomical regions derived manually or using the trained 3D-UNet model for the segmentation of renal parenchyma (red) and renal sinus fat (yellow) from the hold-out test of dual-echo gradient echo (GRE) images.


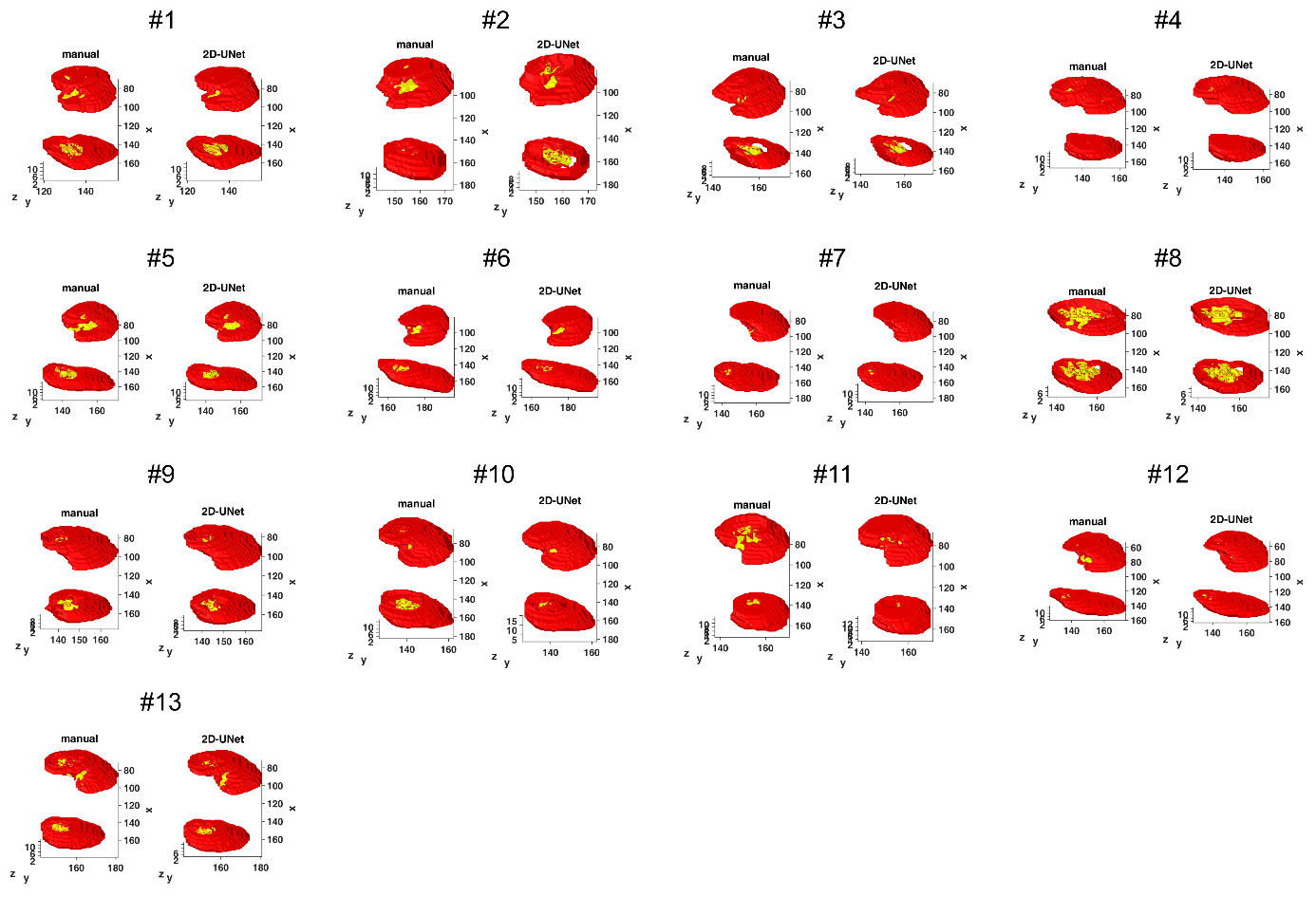


Fig. 2: Illustration of manual and automatic segmentation from whole-body MRI

Display of 3D surface representations of anatomical regions derived manually or using the trained 3D-UNet model for the segmentation of renal parenchyma (red) and renal sinus fat (yellow) from the hold-out test of whole-body MRI.


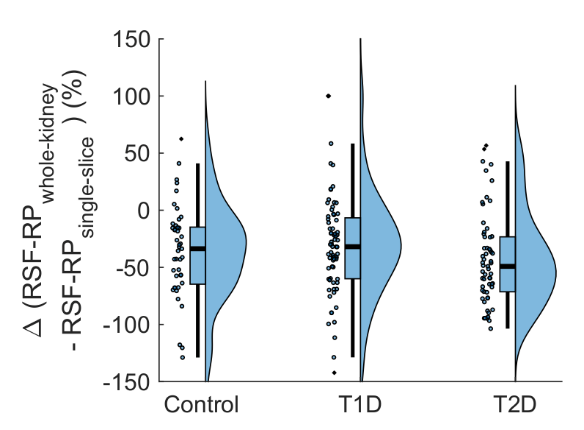


Fig. 3: Comparison between whole-kidney and single-slice approach for RSF-RP ratio analysis

The half-violin plots and the corresponding scatter and boxplots display the percentage difference between the renal sinus fat-to-renal parenchyma (RSF-RP) ratio calculated using a whole-kidney and single-slice approach. Median percentage differences are displayed (black lines).


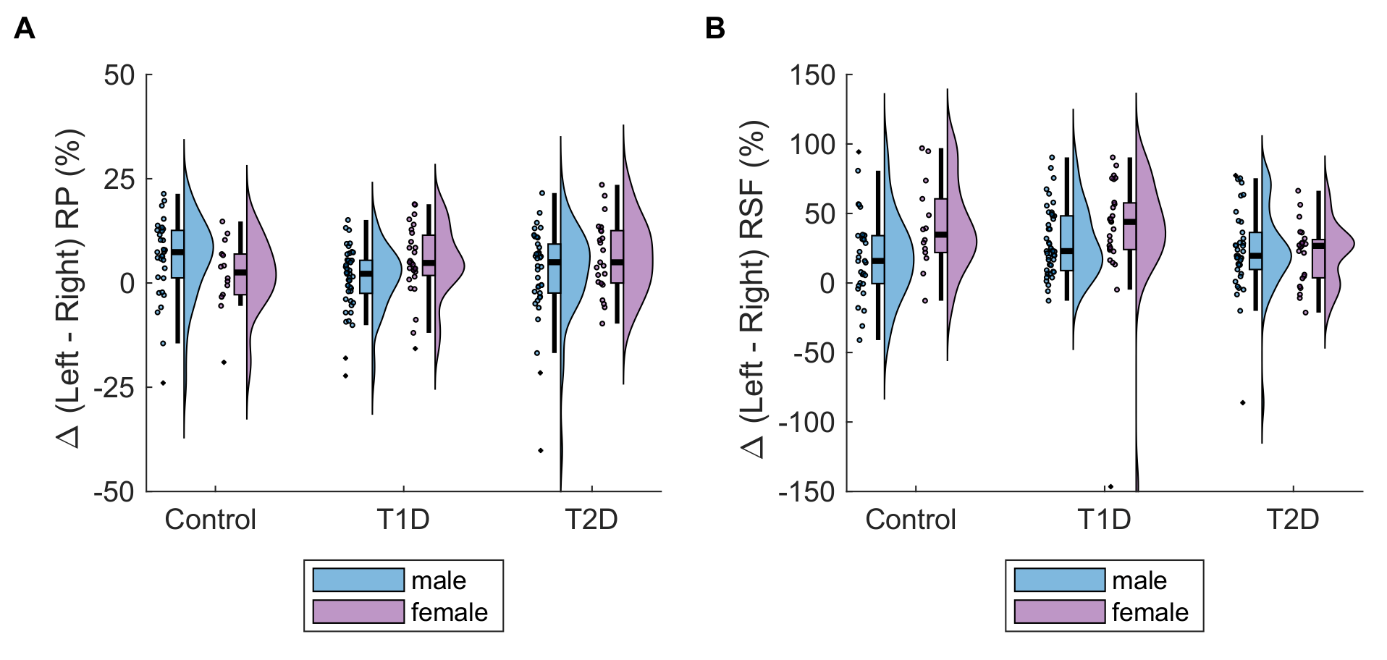


Fig. 4 Comparison of left and right kidney structures across gender and glycaemic status

The half-violin plots and the corresponding scatter and boxplots display the percentage difference between the volume of the left and the right kidney for (**A**) the renal parenchyma (RP) and (**B**) renal sinus fat (RSF).


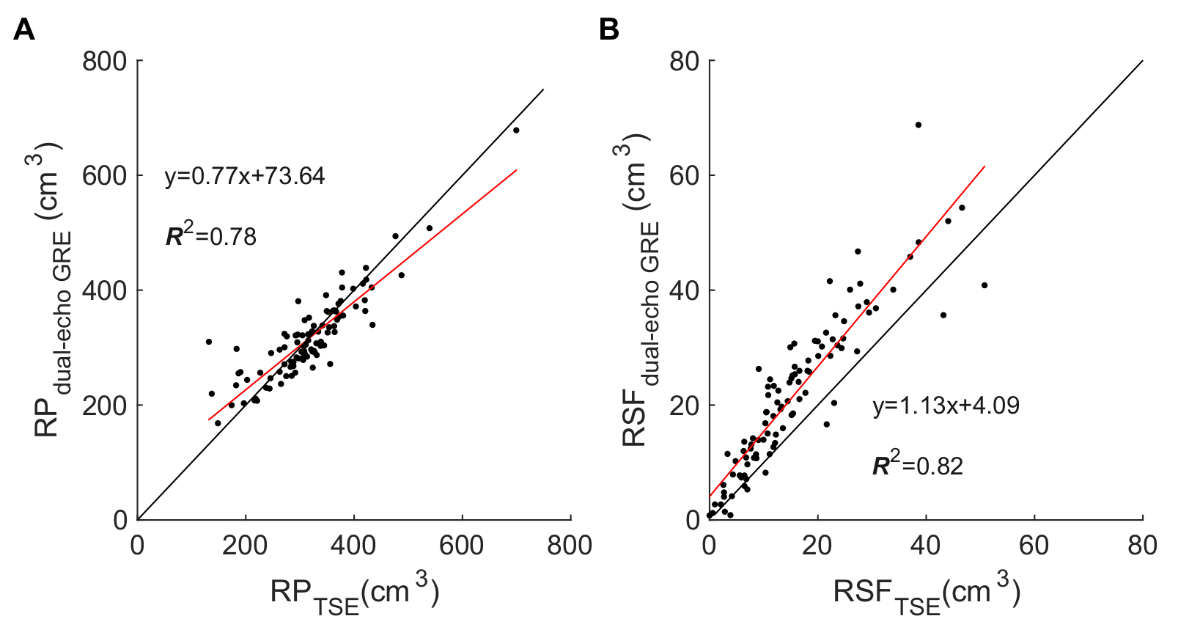


Fig. 5: Comparison of renal parenchyma and renal sinus fat between high-resolution abdominal GRE and whole-body TSE images with interslice-gaps

The scatter plots display the estimates of (**A**) renal parenchyma (RP) (cm^3^) and (**B**) renal sinus fat (RSF) (cm^3^) calculated from the dual-echo gradient-echo (GRE) images against the respective values from the same person obtained from the analysis of turbo-spin echo (TSE) images acquired with interslice-gaps. Linear regression (red line) and identity lines (back line) as well as the coefficients of determination (*R*^2^) are displayed.


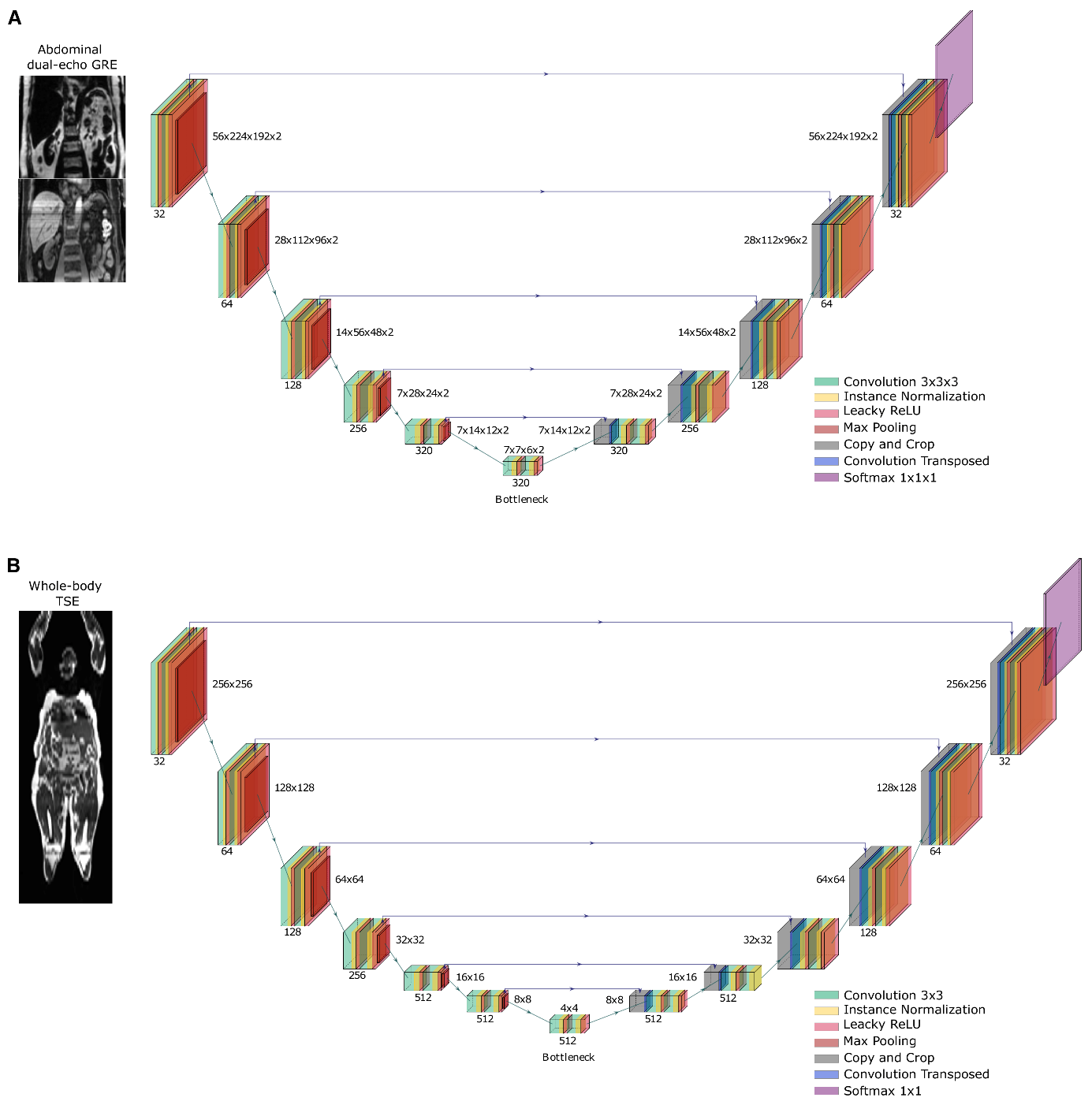


Fig. 6: U-Net architectures for abdominal GRE and whole-body TSE images

Display of the coronal view of the (**A**) abdominal dual-echo gradient echo (GRE) and (**B**) whole-body turbo spin-echo (TSE) MRI protocols and their respective 3D- and 2D U-shaped convolutional neural networks, which were optimized using a nnU-Net framework. ​The networks consist of a decoder-encoder structure with skip connections to preserve spatial information. For each level, two sequential blocks of convolution-instance normalization-activation layers were applied following max-pooling. A softmax multi-class function is used to reduce the number of feature maps to the number of output classes.


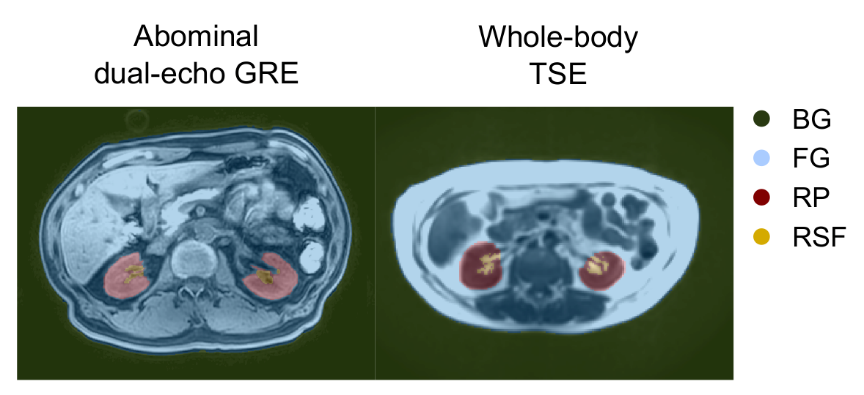


Fig. 7: Annotations for renal parenchyma and sinus fat regions

Representative axial MR images with overlaid manual references of renal parenchyma (red), renal sinus fat (yellow). The areas for foreground (blue) and background (green) were obtained after image post-processing and additionally given to the respective networks during the training.

# Supplementary Information Tables

Table 1: Summary of the parameters used for abdominal dual-echo GRE and whole-body MR image acquisition

|  | General MRI parameters | |
| --- | --- | --- |
|  | **abdominal**  **dual-echo GRE** | **whole-body**  **TSE** |
| TR/TE(s) (ms) | 3.76/1.32, 2.40 | 400/38 |
| FA (°) | 10 | 90 |
| Slice thickness (mm) | 2 | 10 |
| Interslice-gap (mm) | - | 10 |
| FoV (mm) | 375×306×250 |  |
| Matrix size | 252×170×125 | 256×256 |
| BW (Hz) | 1240 | 787 |
| ETL | - | 7 |
| SENSE factor | 2 | - |
| *BW: Bandwidth*  *ETL: Echo Train Length*  *GRE: Gradient-Echo*  *FA: Flip Angle*  *FoV: Field of View*  *TE: Time of Echo*  *TR: Time of Repetition*  *TSE: Turbo-Spin Echo*  *SENSE: Sensitivity Encoding* | | |

Table 2: Left and right kidney quantification from whole-kidney analysis of dual-echo GRE images

|  | *Male* | | | | | |
| --- | --- | --- | --- | --- | --- | --- |
|  | **Control**  **(n=31)** | | **T1D**  **(n=45)** | | **T2D**  **(n=38)** | |
|  | **RP** | **RSF** | **RP** | **RSF** | **RP** | **RSF** |
| Left kidney (cm^3^) | 160.1  ±36.3 | 15.7  ±8.4 | 168.5  ±35.8 | 12.7  ±6.3 | 174.9  ±36.2 | 13.7  ±8.0 |
| Right kidney (cm^3^) | 149.6  ±31.2 | 13.4  ±8.2 | 166.5  ±37.4 | 9.9  ±6.5 | 170.3  ±36.8 | 11.0  ±8.0 |
| Δ (%) | 5.8  ±9.8 | 19.1  ±30.1 | 1.1  ±7.6 | 29.1  ±24.3 | 2.4  ±11.3 | 23.2  ±30.6 |
|  | ***Female*** | | | | | |
|  | **Control**  **(n=14)** | | **T1D**  **(n=27)** | | **T2D**  **(n=23)** | |
|  | **RP** | **RSF** | **RP** | **RSF** | **RP** | **RSF** |
| Left kidney (cm^3^) | 152.6  ±36.1 | 11.7  ±7.1 | 151.3  ±41.4 | 9.9  ±6.6 | 143.8  ±25.1 | 12.7  ±8.5 |
| Right kidney (cm^3^) | 149.3  ±36.2 | 8.0  ±7.0 | 142.3  ±36.2 | 6.3  ±5.2 | 134.7  ±25.8 | 10.6  ±8.1 |
| Δ (%) | 2.1  ±8.5 | 40.8  ±31.6 | 5.2  ±8.5 | 36.9  ±44.0 | 6.3  ±8.7 | 20.9  ±21.9 |
| *Note: values are reported as mean±standard deviation (SD)*  *RP: Renal Parenchyma*  *RSF: Renal Sinus Fat*  *T1D: Type 1 Diabetes*  *T2D: Type 2 Diabetes* | | | | | | |
